# Roles for NHERF1 and NHERF2 on the Regulation of C3a Receptor Signaling in Human Mast Cells

**DOI:** 10.1371/journal.pone.0051355

**Published:** 2012-12-20

**Authors:** Hariharan Subramanian, Kshitij Gupta, Hydar Ali

**Affiliations:** Department of Pathology, School of Dental Medicine, University of Pennsylvania, Philadelphia, Pennsylvania, United States of America; Medical School of Hannover, United States of America

## Abstract

**Background:**

The anaphylatoxin C3a binds to the G protein coupled receptor (GPCR, C3aR) and activates divergent signaling pathways to induce degranulation and cytokine production in human mast cells. Adapter proteins such as the Na^+^/H^+^ exchange regulatory factor (NHERF1 and NHERF2) have been implicated in regulating functions of certain GPCRs by binding to the class I PDZ (PSD-95/Dlg/Zo1) motifs present on their cytoplasmic tails. Although C3aR possesses a class I PDZ motif, the possibility that it interacts with NHERF proteins to modulate signaling in human mast cells has not been determined.

**Methodology/Principal Findings:**

Using reverse transcription PCR and Western blotting, we found that NHERF1 and NHERF2 are expressed in human mast cell lines (HMC-1, LAD2) and CD34^+^-derived primary human mast cells. Surprisingly, however, C3aR did not associate with these adapter proteins. To assess the roles of NHERFs on signaling downstream of C3aR, we used lentiviral shRNA to stably knockdown the expression of these proteins in human mast cells. Silencing the expression of NHERF1 and NHERF2 had no effect on C3aR desensitization, agonist-induced receptor internalization, ERK/Akt phosphorylation or chemotaxis. However, loss of NHERF1 and NHERF2 resulted in significant inhibition of C3a-induced mast cell degranulation, NF-κB activation and chemokine production.

**Conclusion/Significance:**

This study demonstrates that although C3aR possesses a class I PDZ motif, it does not associate with NHERF1 and NHERF2. Surprisingly, these proteins provide stimulatory signals for C3a-induced degranulation, NF-κB activation and chemokine generation in human mast cells. These findings reveal a new level of complexity for the functional regulation of C3aR by NHERFs in human mast cells.

## Introduction

Cross-linking of high affinity IgE receptors (FcεRI) on mast cells is known to play an important role in allergic and hypersensitive diseases [1]. Fukuoka et al [2] showed that activation of human mast cells via FcεRI results in the secretion of tryptase, which generates sufficient amounts of C3a from C3 to cause mast cell degranulation. They proposed that C3a-induced mast cell activation may play an important role in mediating allergic diseases. Indeed, Shafer et al., [3] recently demonstrated that IgE-mediated passive cutaneous anaphylaxis resulted in local increase in C3a levels and that subsequent activation of C3aR on mast cells contributed to allergic skin response. Not surprisingly, we have shown that C3a causes degranulation and chemokine generation in human mast cells and in transfected RBL-2H3 cells [4]–[6]. However, the mechanisms involved in the regulation of C3aR signaling in mast cells remain poorly defined.

A large number of multi-domain scaffolding proteins, including PDZ (PSD-95/Dlg/Zo1) domain containing proteins, associate with GPCRs [7], [8]. There are three general classes of PDZ domains; class I domain, which recognize the carboxyl terminal motif S-T-X-Φ, (where “Φ” indicates hydrophobic amino acid and “X” indicates any amino acid), class II domain which recognize carboxyl terminal motif Φ-X-Φ and class III domains, which recognize D/E-X-Φ as their preferred carboxyl terminal motif [9]. Na^+^/H^+^ exchanger regulatory factor-1 and 2 (NHERF1, EBP-50, *SLC9A3R1* and NHERF2, TKA-1, *SLC9A3R1*) are two class I PDZ domain adapter proteins that bind to several GPCRs to regulate their signaling [10]. Both NHERF1 and NHERF2 have been shown to bind to parathyroid hormone receptor (PTHR) and β2-adrenergic receptor (β2-AR) via their PDZ binding domains to modulate receptor desensitization and internalization [11]–[15]. NHERF proteins can also regulate GPCR signaling in receptor-independent manner via their association with downstream signaling proteins. For example, NHERF1 and NHERF2 associate with phospholipase C-β to regulate GPCR activation [16], [17]. Moreover, NHERF1 blocks PTH-induced ERK1/2 phosphorylation downstream of PKA via a receptor-independent but Akt-dependent pathway [18].

C3aR is one of the few GPCRs expressed in human mast cells that possess a class I PDZ motif. We therefore postulated that NHERF1 or NHERF2 could associate with C3aR to modulate signaling in mast cells. We have recently utilized lentivirus short hairpin (sh)RNA to stably knockdown the expression of receptors, protein kinases and adapter molecules in human mast cell lines, HMC-1, LAD2 and primary CD34^+^-derived human mast cells to study receptor signaling [19]–[21]. We used a similar approach for the current study and used both human mast cells lines (HMC-1 and LAD2) as well primary human CD34^+^-derived mast cells and show that although NHERF1 and NHERF2 do not interact with C3aR, they play a critical role in modulating C3a-induced degranulation and chemokine production.

## Results

### NHERF1 and NHERF2 are expressed in human mast cells but they do not interact with C3aR

**Figure 1:**
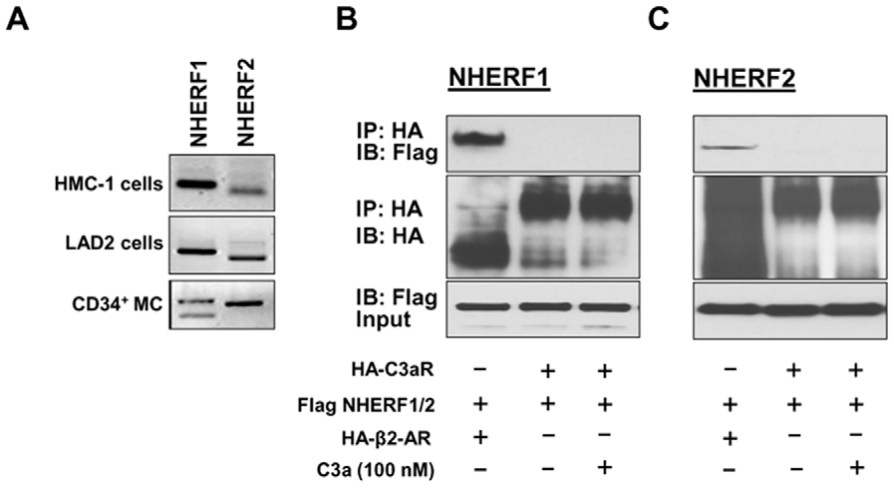
NHERF1 and NHERF2 are expressed in human mast cells but they do not associate with C3aR. (A) NHERF1 and NHERF2 expression was determined in human mast cell lines (HMC-1, LAD2) and CD34^+^ primary human mast cells (CD34^+^ MC) by RT-PCR. Transient transfectants were generated in HEK293 cells expressing HA-tagged C3aR or β2 adrenergic receptor (β2-AR) and (B) Flag-tagged NHERF1 or (C) NHERF2. Cells were exposed to buffer or C3a (100 nM, 37°C for 5 min) as indicated, lysed, immunoprecipitated with anti-HA-antibody, resolved by 10% SDS-PAGE, and transferred onto nitrocellulose membrane. Blots were then probed with anti-Flag antibody to detect NHERF binding to the receptor (top panel) or anti-HA antibody to examine receptor expression (middle panel). Western blotting was performed with anti-Flag antibody on the lysate samples (input) to determine NHERF expression levels (bottom panel). A representative blot from three independent experiments is shown.

NHERF proteins are highly expressed in epithelial cells of the gastrointestinal tract and airways [10], [22], [23]. Since the goal of the present study was to determine the role of NHERFs on regulating C3aR signaling in mast cells, we wanted to first test if these proteins are expressed in human mast cells. We therefore performed reverse transcription PCR on cDNAs obtained from an immature (HMC-1), mature (LAD2), and primary (CD34^+^-derived) human mast cells using primers specific for human NHERF1 and NHERF2. Fig. 1A shows that mRNAs for both NHERF1 and NHERF2 were detected in all three types of human mast cells. To determine if NHERFs physically associate with C3aR, we co-transfected HA-tagged C3aR and Flag-tagged NHERF1 or NHERF2 in HEK 293 cells and performed co-immunoprecipitation experiments. Surprisingly, NHERF1 or NHERF2 failed to interact with C3aR even when the cells were stimulated with C3a (Fig. 1B and C, lane 3, upper panel). It is quite possible that the lack of C3aR-NHERF interaction reflects the experimental condition used for the present study. It has previously been shown that NHERF1 and NHERF2 strongly associated with β2-adrenergic receptor (β2-AR) [11], [24]. We therefore co-expressed HA-tagged β2-AR and Flag-tagged NHERF1 or NHERF2 and performed co-immunoprecipitation study under the exact same condition as was done for C3aR-NHERF interaction. As shown in (Fig. 1B and 1C, lane 1, upper panel), β2-AR co-immunoprecipitated with NHERF1 and NHERF2. This clearly demonstrates that despite the presence of class I PDZ motif at the carboxyl terminus of C3aR, it does not associate with NHERF1 or NHERF2.

### Silencing the expression of NHERF1 and NHERF2 in human mast cells

**Figure 2:**
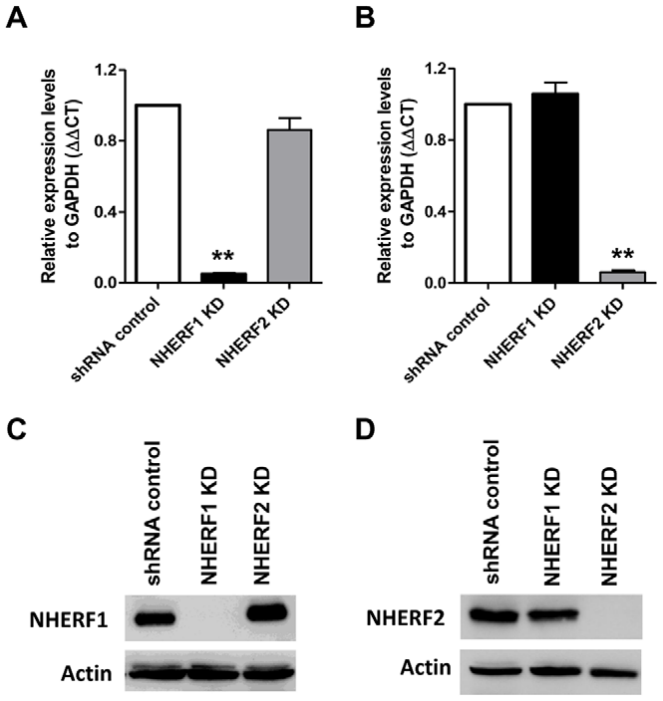
Knockdown of NHERF1 and NHERF2 in HMC-1 cells. Cells were stably transduced with scrambled shRNA control lentivirus or shRNA lentivirus targeted against NHERF1 or NHERF2. Quantitative PCR was performed to assess (A) NHERF1 and (B) NHERF2 mRNA levels in shRNA control and NHERF knockdown (KD) cells. Results are expressed as a ratio of NHERF to GAPDH mRNA levels. Data represent the mean ± SEM from three independent experiments. Statistical significance was determined by unpaired two-tailed *t* test. ** indicates p<0.001. Western blotting was also performed to examine NHERF protein levels. Representative immunoblots of HMC-1 cells with knockdown of (C) NHERF1 and (D) NHERF2 from three different experiments are shown.

NHERF proteins regulate signaling in a variety of cells via their interaction with receptors and downstream signaling proteins [11], [16]–[18]. This suggests that these proteins could regulate C3aR signaling and mediator release in human mast cells. To test these possibilities, we used the Mission shRNA lentivirus system to stably knockdown the expression of NHERF1 and NHERF2 in human mast cells. Although HMC-1, LAD2 and primary CD34^+^ cells endogenously expressed C3aR, we used HMC-1 cells for these initial studies mainly because these cells can be cultured in large quantities and are more amenable to genetic manipulation. Cells were separately transduced with lentivirus containing 5 different shRNA constructs targeting different regions of NHERF1 and NHERF2. For control, we used a scrambled shRNA construct. After transduction and selection with puromycin, quantitative real-time PCR and Western blotting were performed to determine the extent of NHERF knockdown. As shown in Fig. 2A and B, clone 2 (TRCN0000043736) for NHERF1 and clone 1 (TRCN0000043707) for NHERF2 showed >90% decrease in mRNA. Consistent with this observation, Western blotting analyses showed complete absence of these proteins in knockdown cells (Fig. 2C and D). We therefore used these HMC-1 knockdown cells for subsequent experiments.

### NHERF1 and NHERF2 do not regulate C3aR desensitization, internalization, ERK1/2 phosphorylation, Akt phosphorylation or chemotaxis

**Figure 3:**
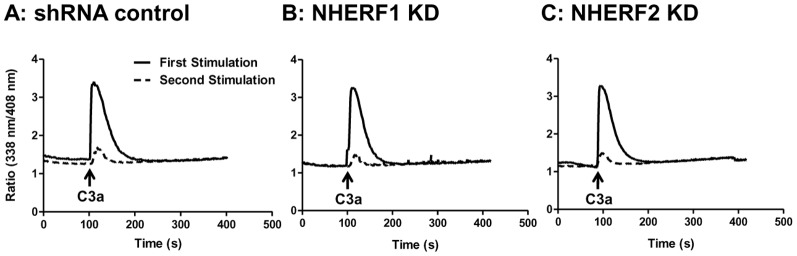
Silencing NHERF1 or NHERF2 has no effect on agonist-induced C3aR desensitization. (A) shRNA control, (B) NHERF1, and (C) NHERF2 KD HMC-1 cells were loaded with Indo-1(1 µM), stimulated with C3a (100 nM) for 5 min and intracellular Ca^2+^ mobilization was determined (solid lines). The cells were washed three times with ice-cold buffer, resuspended in warm buffer and exposed to a second stimulation of C3a (100 nM) and intracellular Ca^2+^ mobilization was again determined (broken lines). Representative traces from at least three separate experiments are shown.

Agonist-induced phosphorylation of C3aR results in β-arrestin-2-mediated receptor desensitization and internalization [20], [25]. NHERF1 associates with PTHR, blocks β-arrestin-2 binding to inhibit desensitization and internalization [26], [27]. By contrast, NHERF1 promotes β-arrestin-2 interaction with the chemokine receptor CCR5 to enhance receptor internalization [28]. These findings suggest that NHERF proteins could regulate C3aR desensitization and internalization in mast cells. We have recently used intracellular Ca^2+^ mobilization assay in HMC-1 cells to determine the roles of GRKs and β-arrestins on C3aR desensitization [19], [20]. We therefore used this assay to determine the roles of NHERF1 and NHERF2 on C3aR desensitization. As shown in Fig. 3A, C3a caused a rapid increase in Ca^2+^ mobilization in shRNA control cells which decayed rapidly to baseline levels in ∼200 sec. The kinetics of this response was not altered in NHERF1 or NHERF2-silenced HMC-1 cells (Fig. 3B and C), indicating that these adapter proteins are not involved in C3aR desensitization. To test further the roles of NHERF1/NHEFR2 on C3aR desensitization, shRNA control or knockdown cells were stimulated with C3a, washed twice before re-exposure to the same concentration of C3a. shRNA control cells demonstrated at least a 80–90% reduction in intracellular Ca^2+^ mobilization indicating that the receptors were desensitized (Fig. 3A). A similar response was also observed in NHERF1 and NHERF2 knockdown cells (Fig. 3B and C). These studies clearly demonstrate that NHERFs do not regulate C3aR desensitization in HMC-1 cells.

**Figure 4:**
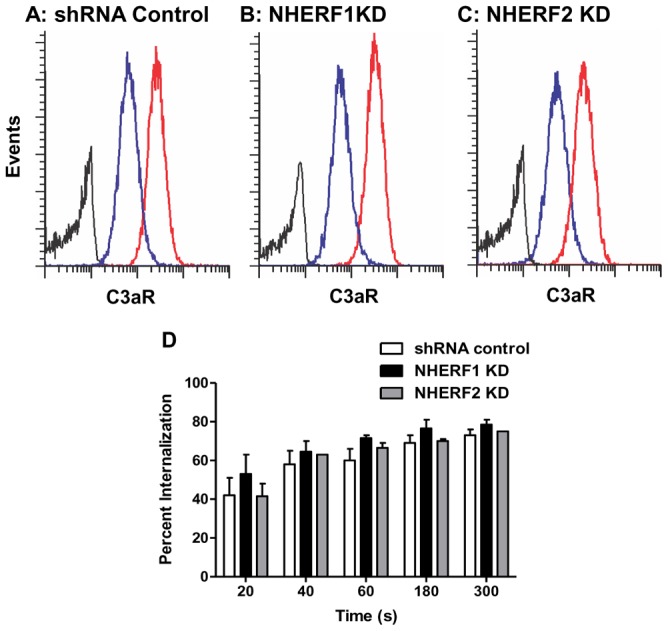
Knockdown of NHERF1 and NHERF2 does not affect C3aR internalization. (A) shRNA control HMC-1 cells (B) NHERF1 knockdown (KD) and (C) NHERF2 KD cells were exposed to buffer or C3a (100 nM) for 5 min. Cells were washed with ice-cold FACS buffer, incubated with a mouse anti-C3aR antibody or an isotype control antibody followed by PE-labeled donkey anti-mouse IgG antibody and analyzed by flow cytometry. Representative histograms plots from an internalization experiment following exposure to buffer (red line) or C3a (blue line) in shRNA control and NHERF KD cells are shown. (D) shRNA control, NHERF1 KD and NHERF2 KD cells were exposed to C3a for different time periods and receptor internalization was determined as described above. Internalization is expressed as the percentage loss of C3aR following exposure to C3a. Data represent the mean ± SEM from three experiments.

To investigate the role of NHERFs on agonist-induced C3aR internalization, shRNA control, NHERF1 and NHERF2 knockdown cells were exposed to buffer or C3a (100 nM) for different time intervals (20–300 sec) and loss of cell surface receptors was determined by flow cytometry. In shRNA control cells, C3a caused a robust internalization of its receptors with up to 70% of the receptors internalized within 5 min (Fig. 4A and D). However, consistent with our finding that NHERFs does not associate with C3aR (Fig. 1), NHERF1 and NHERF2-silenced cells did not demonstrate a significant difference in receptor internalization as compared to control cells at any of the time points tested (Fig. 4). These findings demonstrate that unlike the situation with PTHR and CCR5, NHERF proteins do not regulate C3aR desensitization or internalization.

**Figure 5:**
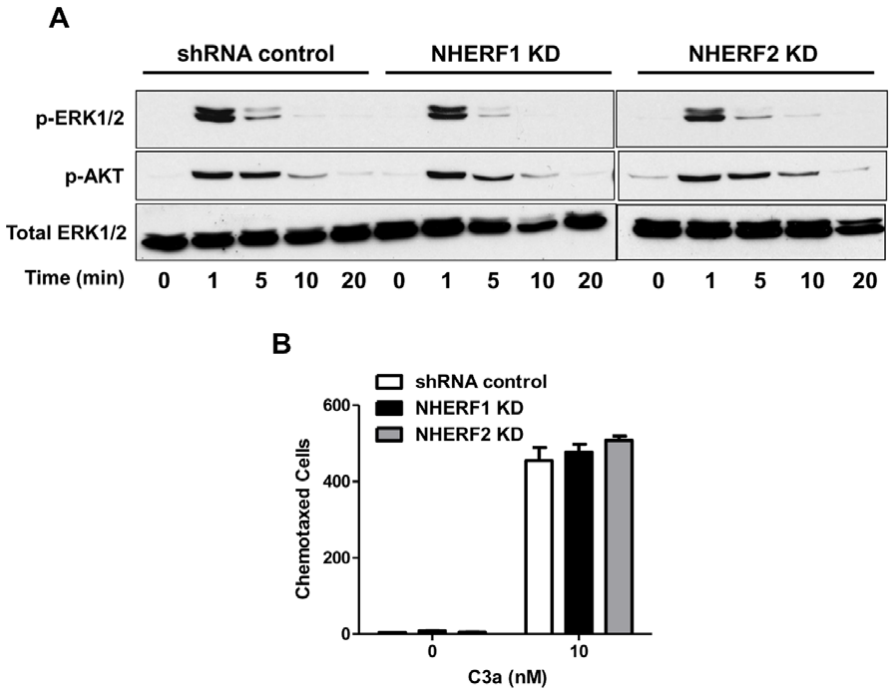
Knockdown of NHERF1 and NHERF2 does not affect C3a-induced ERK/Akt phosphorylation, or chemotaxis. (A) shRNA control and NHERF KD HMC-1 cells were stimulated with C3a (100 nM) for indicated time intervals. Cell lysates were separated on SDS-PAGE and blots were probed with anti-phospho-ERK1/2 antibody followed by anti-rabbit IgG-HRP antibody. Immunoreactive band were visualized by SuperSignal West Femto maximum sensitivity substrate. The blots were also stripped and reprobed with anti-phopho-Akt and anti-ERK1/2 antibody followed by anti-rabbit IgG-HRP. A representative immunoblot from three similar experiments is shown. (B) shRNA control and NHERF KD HMC-1 cells were allowed to chemotax to C3a (10 nM, 37°C for 3 h). Data is represented as the total number of chemotaxed cells in the lower chamber. Bar graphs represent the mean ± SEM from three independent experiments.

NHERF proteins have been shown to associate with a number of signaling proteins to regulate Akt and ERK phosphorylation independently of their interaction with PDZ motif containing GPCRs. Thus, NHERF1 associates with phosphatase and tensin homolog (PTEN), a major regulator of phosphatidylinositol 3-kinases (PI3K) pathway, to enhance Akt phosphorylation [29]. By contrast, NHERF1 blocks PTH-induced ERK1/2 phosphorylation downstream of PKA via a receptor-independent but Akt-dependent pathway [18]. NHERF1 and NHERF2 also associate with phospholipase C-β to modulate ERK1/2 phosphorylation [16], [17]. We therefore expected NHERF proteins to modulate C3a-induced ERK1/2 and or Akt phosphorylation in mast cells. We found that in shRNA control cells, C3a caused a transient ERK1/2 phosphorylation that peaked between 0–5 min and returned to basal levels thereafter. However, silencing NHERF1 or NHERF2 expression had no effect on C3a-induced ERK1/2 phosphorylation (Fig. 5A). Similar results were also observed for C3a-induced Akt phosphorylation in HMC-1 cells (Fig. 5A). In neutrophils, the chemokine receptor CCR2 forms a signaling complex with NHERF and phospholipase Cβ2 and that disruption of this complex leads to decreased neutrophil migration [30]. Given that C3a is a potent mast cells chemoattractant [31], [32], we examined the role of NHERFs in regulating C3a-induced mast cell chemotaxis. C3a (10 nM) caused chemotaxis of HMC-1 cells in shRNA cells and there was no significant difference in this response in NHERF1 or NHERF2-silenced cells (Fig. 5B).

### NHERF1 and NHERF2 promote C3a-induced mast cell degranulation, NF-κB activation and chemokine CCL4 generation

**Figure 6:**
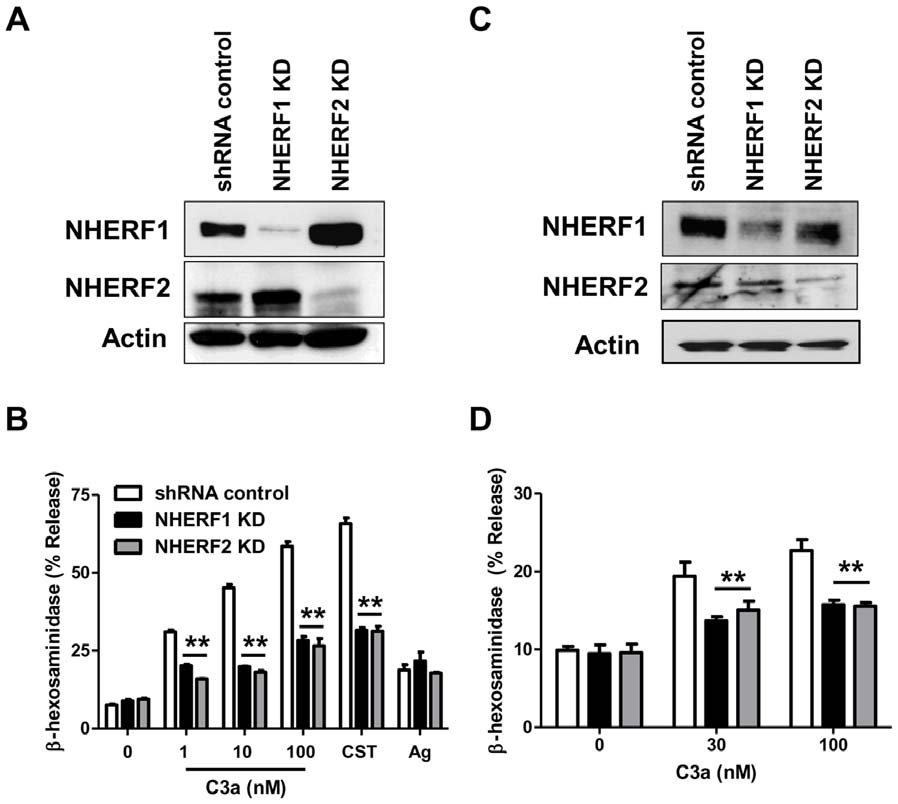
NHERF1 and NHERF2 contribute to C3a-induced degranulation response in LAD2 and CD34^+^ primary human mast cells. LAD2 or CD34^+^ primary human mast cells were stably transduced with scrambled shRNA control lentivirus or shRNA lentivirus targeted against NHERF1 or NHERF2. Western blotting was performed to assess NHERF expression levels in shRNA control and NHERF KD (A) LAD2 and (C) CD34^+^ primary human mast cells. A representative blot from three independent experiments is shown. shRNA control or NHERF KD (B) LAD2 mast cells or (D) CD34^+^ mast cells were stimulated with indicated concentrations of C3a or CST (100 nM) and percent degranulation (β-hexosaminidase release) was determined. LAD2 cells were also stimulated by NP-specific IgE/NP-BSA (Ag) as a non-GPCR control. Data are mean ± SEM of three experiments. Statistical significance was determined by two-way ANOVA with Bonferroni's post test. ** indicates p<0.001.

Our next goal was to determine if NHERF1 and NHERF2 regulate C3a-induced mast cell degranulation. HMC-1 is an immature mast cell line that shows little or no degranulation response to C3a. LAD2 is a mature mast cell line that expresses C3aR and responds to C3a for degranulation [4], [6], [33]. We therefore silenced the expression of NHERF1 and NHERF2 in LAD2 cells. As in HMC-1 cells, lentiviral shRNA induced ∼90% knockdown of the NHERF1 and NHERF2 in LAD2 mast cells (Fig. 6A). Surprisingly, knockdown of NHERF1 and NHERF2 resulted in a significantly decreased degranulation response to varying doses of C3a (Fig. 6B). This effect was specific for C3a as silencing the expression of NHERFs had little or no effect on antigen-IgE-mediated response. We have recently identified a novel GPCR, MrgX2 that is expressed in human mast cells [21], [33]. Cortistatin-14 (CST), a ligand for MrgX2, induced substantial degranulation in shRNA control LAD2 cells and this response was significantly inhibited in NHERF1 and NHERF2 knockdown cells. These data suggest that C3aR and MrgX2 share a common pathway for inducing degranulation, which is different from IgE-mediated response. We have previously shown that both primary cultures of human CD34^+^-derived mast cells and the LAD2 mast cell line express C3aR and respond to C3a for degranulation [4], [6]. To confirm the biological relevance of studies with LAD2 mast cells, we silenced the expression of NHERF1 and NHERF2 in CD34^+^-derived primary human mast cells. Unlike the situation in LAD2 cells (Fig. 6A), we were able to silence the expression of NHERF1 and NHERF2 in CD34^+^-derived primary human mast cells by ∼50% (Fig. 6C). More importantly, even this knockdown efficiency was sufficient to cause a significant reduction in C3a-induced degranulation (Fig. 6D).

**Figure 7:**
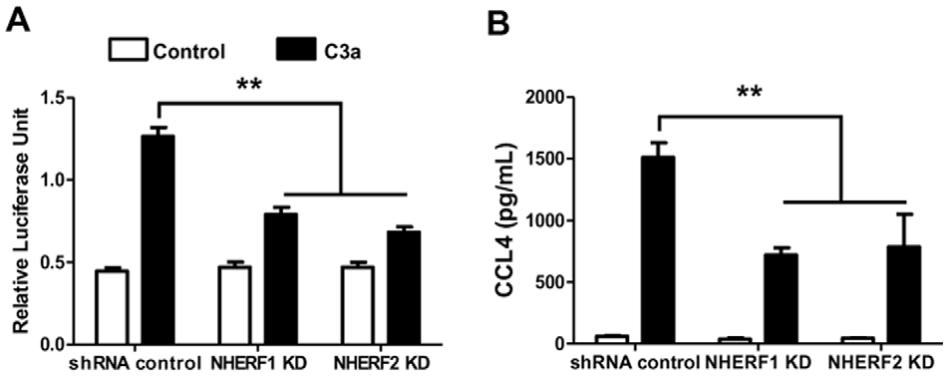
NHERF1 and NHERF2 contribute to C3a-induced NF-κB activation and chemokine CCL4 generation. shRNA control, NHERF1 KD or NHERF2 KD HMC-1 cells were transiently transfected with NF-κB luciferase reporter gene construct along with C3aR. (A) Cells were stimulated with C3a (100 nM for 6 hr) and NF-κB-dependent transcriptional activity was determined by luciferase activity assay. Data is presented as relative luciferase activity normalized to Renilla luciferase activity. (B) Control or NHERF KD cells were stimulated with C3a (100 nM for 6 h) and CCL4 production was determined from the supernatant by ELISA. Data shown are mean ± SEM of three experiments performed in triplicate. Statistical significance was determined by two-way ANOVA with Bonferroni's post test. ** indicates p<0.001.

We next sought to determine the roles of these PDZ domain proteins on C3a-induced NF-κB luciferase activity in mast cells. Although HMC-1 cells, LAD2 mast cells and CD34^+^-derived primary mast cells express C3aR and respond to C3a for Ca^2+^ mobilization, C3a does not induce NF-κB activation in HMC-1 cells unless the cells are transfected with exogenous receptor [4], [20], [34]. Therefore, we transfected C3aR along with NF-κB luciferase constructs in shRNA control and NHERF-silenced cells and performed NF-κB luciferase and chemokine release assays. C3a induced NF-κB luciferase activity in shRNA control HMC-1 cells (Fig. 7A). Interestingly, there was a significant reduction in NF-κB luciferase activity in NHERF knockdown cells when compared to shRNA control cells. Given that NF-κB plays an important role in the generation of proinflammatory cytokines, we tested the effects of NHERF1 and NHERF2 knockdown on C3a-induced chemokine CCL4 production. We found that C3a-induced CCL4 production was significantly decreased in NHERF knockdown cells (Fig. 7B).

## Discussion

The roles of NHERF proteins in regulating receptor desensitization, internalization and signaling have been studied extensively for GPCRs such as β2-AR, PTHR and opioid receptors [10]. Most of these studies have utilized NHERF-null renal tubule cells or transfected rat osteosarcoma ROS 17/2.8 (ROS), opossum kidney (OK), PS120, and HEK 293 cells [12], [13], [35]–[38]. For the present study, we utilized lentivirus shRNA to stably knockdown the expression of NHERF1 and NHERF2 in human mast cell lines; HMC-1 cells, LAD2 and primary CD34^+^-derived human mast cells that endogenously express C3aR. Using this approach, we have uncovered novel roles of NHERF1 and NHERF2 on C3a-induced degranulation, NF-κB activation and chemokine generation in the absence receptor desensitization, internalization, ERK1/2 and Akt phosphorylation.

NHERF1 and NHERF2 are class I PDZ domain proteins that associate with the carboxyl terminal of several GPCRs. Class I PDZ proteins interact with ligands terminating in the sequence [S/T]- X-Φ, where X is promiscuous and Φ is a hydrophobic residue, generally Leu but also Ile, Val, or Met. Our results indicate that although C3aR possess a class I PDZ protein binding motif in its carboxyl terminus (S-T-T-V), NHERF proteins do not interact with the receptor. It has been proposed that in addition to the class I PDZ motif, NHERFs seems to additionally favor [D/E] at position -3 [10]. Hall *et al.*, have reported that the sequence D-T-R-L is favored for binding by NHERFs [11]. However, NHERFs also bind to N-K-P-V and S-V-G-L sequences in opioid and CCR5 receptors [28], [39]. Thus, the consensus binding motif for NHERFs seems to vary and may require additional binding sites upstream or downstream of the PDZ binding site. Accordingly, it has been proposed that selectivity for PDZ protein-receptor interaction may be influenced by upstream amino acids at positions 5 and/or 7 [40]. Thus, the inability of NHERFs to associate with the C3aR probably reflects the lack of these upstream structural determinants required for NHERF1/2 binding.

In addition to GPCRs, NHERF proteins associate with a variety of downstream signaling proteins to regulate receptor internalization, ERK1/2 phosphorylation, Akt phosphorylation [15], [18], [29]. However, the findings in the present study that NHERF1 and NHERF2 have no effect on these responses but promote mast cell degranulation and chemokine production clearly demonstrate that these proteins have distinct functions in human mast cells. The mechanism by which NHERF1 promotes mast cell degranulation remains unknown. NHERF1 contains 31 Ser and 9 Thr residues, which make up roughly 12% of the molecule. Hall *et al.*, [41] showed that GRK6 interacts with PDZ motif of NHERF1 to promote its phosphorylation at Ser289 [41]. In our recently published work, we have shown that, as for NHERF1, silencing the expression of GRK6 has no effect on C3a-induced Ca^2+^ mobilization but blocks degranulation [19]. Given that GRK6 associates with and phosphorylates NHERF1 (at Ser289), it is possible that this modification provides a mechanism for C3a-induced mast cell degranulation. Interestingly, PTH causes protein-kinase C (PKC)-mediated phosphorylation of NHERF1 at Ser77 (PDZ1 domain) and this modification regulates PTH action [22], [42]–[44]. PKC also phosphorylates NHERF1 at Ser162 (PDZ2 domain) and Ser337/338 (C-terminus) [45]. It is noteworthy that C3a-induced mast cell degranulation requires PKC activation and PKCα binds to NHERF1 through PDZ domain [5], [46]. It is therefore possible that C3aR, PKCα and NHERF1 form a signaling complex to promote phosphorylation of downstream signaling proteins to support degranulation. An interesting finding of the present study was that both NHERF1 and NHERF2 are involved in promoting mast cells degranulation. It is noteworthy that NHERF1 dimerizes with NHERF2 [10]. This suggests that formation of a complex between NHERF1 and NHERF2 may promote C3a-induced mast cell degranulation and that silencing the expression of either protein leads to inhibition of the response. These possibilities will be the subject of future investigations.

Another interesting finding of the present study was that knockdown of NHERF1 and NHERF2 decreased C3a-induced NF-κB activation and chemokine production in human mast cells. However, the molecular mechanism via which NHERF proteins promote chemokine gene expression is unknown. Na^+^/H^+^ exchanger 3 (NHE3) is a member of a group of integral transmembrane proteins that is regulated by NHERF [47]. It has been proposed that in monocytes and macrophages, NHEs are rapidly activated by inflammatory stimulus such as IL-1, TNF-α and LPS which leads to the production of a variety of cytokines [48]–[50]. In conjunction with this hypothesis, Németh *et al.*, [48] have demonstrated that inhibition of NHEs suppressed IL-12, MIP-1α and MIP-2 production by LPS-activated macrophages. In a subsequent study they showed that this inhibition of NHEs suppressed both the inhibitory IκB degradation and NFκB-DNA binding activity leading to a decreased activation of NF-κB and a substantial inhibition in cytokine production [51]. Since NHERFs regulate NHE3 it is tempting to propose that C3a-induced NF-κB activation and cytokine production occurs via a NHE3-dependent pathway and silencing of NHERFs abrogates this pathway. Another possibility is that lack of NHERFs disrupts the cytoskeletal association of actin with the ERM proteins and NHE3 and this change in the actin microfilament organization mediates reduced NF-κB activation and cytokine generation. Whether this or other mechanism(s) participate on the effect of NHERFs on C3a-induced responses in mast cells remains to be determined.

In summary, the current study demonstrates that NHERF1 and NHERF2 promote degranulation and chemokine production, the two critical responses of human mast cells following activation via C3a. Thus, we provide the first demonstration that the PDZ proteins, NHERF1 and NHERF2 may act as novel regulators of C3aR signaling in human mast cells. Since C3a has been reported to potentiate IgE-mediated allergic responses *in vivo*, targeting these adapter proteins in human mast cells may serve as a valuable therapeutic for allergic disorders.

## Materials and Methods

### Materials

Mission shRNA bacterial glycerol stocks for NHERF1 and NHERF2 were purchased from Sigma Life Sciences (St. Louis, MO). Indo-1 AM was from Molecular Probes (Eugene, OR). All tissue culture reagents were purchased from Invitrogen (Gaithersburg, MD). Anti-human C3aR was obtained from Santa Cruz Biotechnology (Santa Cruz, CA), PE-labeled donkey anti-mouse IgG was purchased from eBioscience (San Diego, CA). All recombinant human cytokines were purchased from Peprotech (Rocky Hill, NJ). Rabbit anti-ERK1/2, anti-phospho-ERK1/2 and anti-phospho Akt antibodies were purchased from Cell Signaling (Beverly, MA). SuperSignal® West Femto Maximum Sensitivity Substrate and HRP labeled Goat anti-rabbit IgG were from Thermo Scientific (Rockford, IL). Purified C3a was obtained from Complement Tech, Inc. (Tyler, TX). CCL4 ELISA kit was purchased from R&D Systems (Minneapolis, MN). Amaxa cell transfection kits and reagents were purchased from Lonza (Gaithersburg, MD). Anti Flag antibody (M2) and anti HA (HA-7) agarose beads were purchased from Sigma-Aldrich (St. Louis, MO). Cortistatin-14 (CST) was obtained from American Peptide (Vista, California).

### Mast cell culture

HMC-1 cells were cultured in Iscove's modified Dulbecco's medium (IMDM) supplemented with 10% FCS, L-glutamine (2 mM), penicillin (100 IU/mL) and streptomycin (100 µg/mL) [34]. LAD2 cells were maintained in complete StemPro-34 medium supplemented with 100 ng/mL rhSCF [52]. To obtain primary mast cells, human CD34^+^ progenitors (Hutchinson Cancer Research Center, Seattle, WA) were cultured in StemPro-34 medium supplemented with L-glutamine (2 mM), penicillin (100 IU/ml), streptomycin (100 µg/ml), rhSCF (100 ng/ml), rhIL-6 (100 ng/ml) and rhIL-3 (30 ng/ml) (first week only). Hemidepletions were performed weekly with media containing rhSCF (100 ng/ml) and rhIL-6 (100 ng/ml) (32, 56). Cells were used for experiments after 7–10 weeks in culture [53].

### Co-immunoprecipitation

HEK293 cells transiently transfected with 4 µg of cDNA encoding either the HA-tagged C3aR or HA-tagged β2-AR along with 2 µg of Flag-tagged NHERF were lysed, and clarified by centrifugation. HA-C3aR was immunoprecipitated by addition of anti-HA agarose beads (Sigma) with agitation at 4°C for 4 h. Anti-HA immunocomplexes were washed twice in RIPA lysis buffer and once with ice-cold PBS. Whole-cell lysates and HA affinity matrix immunocomplexes were transferred to nitrocellulose membranes for immunodetection of Flag-NHERF and the C3aR. The C3aR was detected with a 1∶1,000 dilution of mouse anti-HA (12CA5) antibody (Boehringer Mannheim) and Flag-tagged NHERF was detected with a 1∶1,000 dilution of mouse anti-Flag monoclonal antibody (M2). The secondary antibody used in both the cases was a 1∶4,000 dilution of a HRP-conjugated anti-mouse polyclonal antibody (Santa Cruz Biotechnology).

### Lentivirus and stable transduction of shRNAs in mast cells

NHERF1 and NHERF2 targeted shRNAs in lentiviral plasmids were purchased from Sigma-Aldrich. The clone that gave the highest knockdown efficiency (TRCN0000043736 for NHERF1 and TRCN0000043707 for NHERFF2) was used. A scrambled control non-target vector (SHC002) which does not bind to any known human mRNAs was also purchased from Sigma-Aldrich. Lentivirus generation was performed according to the manufacture's manual. Cell transduction was conducted by mixing 1.5 ml of viral supernatant with 3.5 ml of HMC-1 or LAD2 (5×10^6^ cells) or CD34^+^-mast cells (3×10^6^ cells). Eight hours post-infection, medium was changed to virus-free complete medium, and antibiotic (puromycin, 2 µg/ml, Sigma) selection was initiated 16 h later. Cells were analyzed for NHERF1 or NHERF2 knockdown and used for subsequent assays 4 days following initiation of puromycin selection [19], [20].

### Reverse transcription PCR and quantitative PCR

Total RNA was extracted from mast cells using TRIZOL, treated with DNase I and reverse transcribed to cDNA using first strand cDNA synthesis kit (GE). The primers used for human NHERF1 were: forward 5′ TACAGAAGGAGAACAGTCGTGAAGC and reverse 5′ GCCAGGGAGATGTTGAAGTCTAGG. The primers used for human NHERF2 were: forward 5′ CCGACAAGGACACTGAGGATGG and reverse 5′ CGCTTGTTGACTCGCATGGC. For quantitative PCR, gene expression was analyzed using real time PCR with Taqman® Fast Universal PCR Master Mix on a Taqman 7500 Fast Real-Time PCR System (Applied Biosystems, Foster City, CA). Taqman probes for hGAPDH and hNHERFs were used for real time PCR to analyze the knockdown efficiency. The amplification conditions were as follows: initial denaturation at 95°C for 20 sec, followed by 40 cycles of amplification: 95°C for 3 sec, 60°C for 30 sec. Analysis was performed according to ΔΔ-Ct method.

### C3a Receptor desensitization

Receptor desensitization assay based on Ca^2+^ mobilization was determined as described previously [20]. Briefly, 1×10^6^ HMC-1 cells were washed twice with buffer (119 mM NaCl, 5 mM KCl, 25 mM HEPES, 5.6 mM Glucose, 0.4 mM MgCl_2_, 1 mM CaCl_2_) containing 1 mg/ml BSA and incubated with 1 µM of Indo-1 for 30 min in dark. Cells were then washed and resuspended in 1.5 ml of the same buffer and time course of Ca^2+^ mobilization (0–5 min) was determined using Hitachi F-2500 Fluoro spectrophotometer (San Jose, CA) with an excitation wavelength of 355 nm and an emission wavelength of 410 nm. Cells were removed from the cuvette, washed twice and Ca^2+^ mobilization to a subsequent exposure of C3a (100 nM) was determined [19].

### Receptor Internalization

shRNA control and NHERF knockdown HMC-1 cells (2.5×10^5^) were stimulated with or without C3a (100 nM) at 37°C. Cells were washed, resuspended in 50 µl of ice-cold FACS buffer (PBS containing 2% FBS), and stained with C3aR antibody or isotype control (2 µl) on ice for 1 h. After washing twice with ice-cold FACS buffer, cells were labeled with Phycoerythrin (PE)-labeled donkey anti-mouse (1.5 µl) secondary antibody and incubated on ice for 1 h. Cells were washed twice and fixed in 300 µl of 2% formaldehyde. The samples were acquired and analyzed on a BD LSR II flow cytometer (BD Biosciences).

### Western blotting

Control and NHERF knockdown HMC-1 cells or LAD2 cells (1×10^6^/ml) or CD34^+^-derived mast cells (0.5×10^6^/ml) were washed twice in ice-cold PBS and lysed with RIPA buffer (150 mM NaCl, 1.0% NP-40, 0.5% Sodium-deoxycholate, 0.10% SDS, 50 mM Tris [pH 8.0], 5 mM EDTA, 10 mM NaF, 10 mM Na-pyrophosphate and protease inhibitor cocktail). Protein bands were separated on 10% SDS PAGE gels and immunoblotted onto nitrocellulose membranes and probed with anti-NHERF1 (Santa Cruz Biotechnolgy, Santa Cruz, CA) or anti-NHERF2 (Sigma-Aldrich) antibodies. Following incubation with the secondary anti-rabbit-HRP antibody, membranes were developed using the SuperSignal® West Femto Maximum Sensitivity chemiluminiscent substrate.

For ERK/Akt phosphorylation, cells were serum starved overnight, washed twice, resuspended in serum free IMDM medium at a concentration of 1×10^6^/ml and stimulated C3a (100 nM) for different time points. Three-fold volume of ice-cold PBS containing 1 mM sodium orthovanadate was added to stop the reaction. Total cell lysate was prepared with RIPA buffer and subsequently analyzed by Western blotting using rabbit polyclonal antibodies for phospho-p44/42 MAPK (pERK1/2) phospho-Akt (pAkt) and p44/42 MAPK (ERK1/2).

### Chemotaxis assay

C3a (10 nM, 30 µl) or buffer was added to the lower wells of a 96-well chemotaxis chamber (8 µm pore size; NeuroProbe, Gaithersburg, MD). HMC-1 cells (0.5×10^6^) were added on top of the membrane of 96-well chemotaxis chamber. After 3 h incubation at 37°C and 5% CO_2_, chemotaxed cells were collected from the lower chambers. Triplicate wells were pooled and the cells were resuspended in thirty microliters of complete IMDM. The chemotaxed cells were counted under a hemocytometer slide and the results are expressed as absolute number of cells that had chemotaxed.

### Degranulation Assay

LAD2 cells or CD34^+^-derived human mast cells (1×10^4^) were seeded into 96-well plates in a total volume of 50 µl of buffer containing 1 mg/ml BSA and exposed to different concentrations of C3a C3a or CST (100 nM). For some experiments, LAD2 cells were pretreated with NP-specific human IgE (AbD serotec, 1 µg/ml) for 16 h prior to stimulation with NP-BSA (Biosearch Technologies, 100 ng/mL). For total β-hexosaminidase release, control cells were lysed in 50 µl of 0.1% Triton X-100. Aliquots (20 µl) of supernatants or cell lysates were incubated with 20 µl of 1 mM p-nitrophenyl-N-acetyl-β-D-glucosamine for 1.5 h at 37°C. The reaction was stopped by adding 250 µl of a 0.1 M Na_2_CO_3_/0.1 M NaHCO_3_ buffer and absorbance measured at 405 nm [19], [21].

### NF-κB luciferase reporter activity and CCL4 chemokine release assay

shRNA control and NHERF knockdown HMC-1 cells (3×10^6^) were co-transfected with NF-κB luciferase reporter gene construct (pNF-kB-LUC and p-Renilla Stratagene, Santaclara, CA) (in a 10∶1 ratio) along with HA-tagged C3aR using Amaxa nucleofector device and Amaxa kit V as per manufacturer's protocol. Six hour post-transfection, medium was replaced with IMDM containing 10% FBS. Following 18 h of incubation, cells were stimulated with C3a (100 nM for 6 h). Cells were then harvested, washed in ice-cold PBS and finally lysed in Promega passive lysis buffer (Dual Luciferase assay kit; Promega, Madison, WI). NF-κB luciferase activity was measured using Turner biosystem 20/20 Luminometer (Promega, Madison, WI). Results expressed have been normalized to Renilla luciferase activity. Chemokine release assay was performed as previously described [19], [20]. CCL4 chemokine levels in the supernatants were quantified by sandwich ELISA according to the manufacturer's protocol.

### Data analysis

The results are expressed as ± S.E.M for the values obtained from at least three independent experiments. GraphPad Prism software (Graph Pad, Version 5.0 San Diego, CA) was used to analyze data for statistical significance. The statistical significance was determined by unpaired two-tailed *t* test, and two-way ANOVA with Bonferroni's post test. A p value <0.05 was deemed significant.
